# Toward Predictive Design of Lignocellulosic Mycelium-Bound Composites: A Process–Structure–Property Framework, Quantitative Synthesis, and Standardization Roadmap

**DOI:** 10.3390/polym18131652

**Published:** 2026-07-02

**Authors:** Musiliu A. Liadi, Tawakalt O. Ayodele, Ibrahim A. Bello, C. Igathinathane, Hammed M. Ademola

**Affiliations:** 1 Environmental and Conservation Sciences Program, North Dakota State University, Fargo, ND 58108, USA; adeyemimusiliu@gmail.com (M.A.L.); tawakalt.ayodele@ndsu.edu (T.O.A.); 2 Department of Agricultural and Biosystems Engineering, North Dakota State University, 1231 Albrecht Boulevard, Fargo, ND 58102, USA; ibrahim.bello@ndsu.edu

**Keywords:** mycelium composites, lignocellulose, biofabrication, sustainability, biomaterials, standardization

## Abstract

Mycelium-bound composites (MBCs) have emerged as a promising class of biofabricated materials that integrate fungal hyphal networks with lignocellulosic substrates to form lightweight, biodegradable structures without synthetic adhesives. Despite rapid growth in the field, the current literature remains fragmented, with inconsistent methodologies and widely varying reported material properties. This review advances the field by moving beyond descriptive synthesis toward a quantitative and conceptual integration of existing studies. We systematically analyze how key fabrication variables—including fungal species, substrate composition, growth conditions, and post-processing parameters—govern density, porosity, and mechanical performance. A process–structure–property (PSP) framework is proposed to combine these relationships and explain discrepancies across studies. We highlight the dominant role of densification and moisture conditioning in determining compressive strength, often outweighing species-level effects. A comparative synthesis of reported data reveals significant variability in compressive strength (0.05–1.2 MPa) and elastic modulus, attributable to inconsistencies in sample preparation, testing protocols, and environmental conditioning. To address this, we identify critical gaps in standardization and propose actionable testing protocols and reporting guidelines for reproducibility. Furthermore, we assess the technology readiness level (TRL) of MBC systems and distinguish between laboratory-scale innovations and commercially viable processes. While hybridization strategies and biofunctional applications offer promising avenues, their maturity varies widely. This work provides a decision-oriented framework for MBC design and a roadmap for transitioning these materials from experimental systems to scalable, standardized, and application-ready biomaterials.

## 1. Introduction

Mycelium-bound composites (MBCs) have emerged as a novel class of biofabricated materials in which fungal mycelium functions as a biological binder, consolidating lignocellulosic substrates into cohesive structures. Over the past decade, research on MBCs has expanded significantly, driven by increasing demand for sustainable materials and circular bioeconomy solutions. Unlike conventional composites that depend on synthetic resins, MBCs derive their structural integrity from a combination of hyphal interlocking, biochemical adhesion, and physical entanglement within plant-based matrices [1,2]. This unique binding mechanism enables the formation of lightweight (typically 50–300 kg/m^3^), porous, and biodegradable materials while avoiding the environmental and health concerns associated with petrochemical adhesives.

MBCs are suitable for non-structural and semi-structural applications as they exhibit a combination of functional properties, including low thermal conductivity (~0.03–0.07 W/m·K), sound absorption capability, and moderate compressive strength (0.05–4 MPa depending on processing) [3,4,5,6]. Consequently, these materials have been explored for packaging (e.g., protective foams replacing expanded polystyrene), thermal insulation panels, acoustic dampening systems, and interior construction elements [4,7]. Commercial efforts by industries such as Ecovative and MycoWorks demonstrate growing industrial interest, with applications ranging from packaging to bio-based leather alternatives [8]. From an environmental perspective, MBCs are biodegradable under controlled composting conditions, typically degrading within weeks to months, depending on density and environmental exposure, thereby offering a viable solution to plastic waste accumulation [9,10].

In addition to technical performance, MBCs align with broader sustainability and economic goals through the valorization of agricultural residues and reduced energy inputs during manufacturing [2,5]. However, public perception and market adoption remain influenced by concerns regarding durability, moisture sensitivity, and scalability, highlighting the need for improved material consistency and performance validation [8,11].

Despite rapid progress, the field remains marked by fragmented knowledge, inconsistent experimental methods, and substantial variability in reported material properties [1,5]. Several recent reviews have provided comprehensive overviews of fabrication processes, fungal species, substrate pretreatment techniques, and application domains [4,6,7,8,9]. Nevertheless, most of these studies adopt a largely descriptive approach, focusing on summarizing existing findings rather than critically synthesizing data or developing predictive frameworks. As a result, challenges regarding the comparability and reproducibility of reported results remain largely unaddressed.

A central limitation across the literature is the lack of: (i) quantitative comparison of mechanical and physical properties across studies; (ii) standardized testing protocols and reporting methodologies; and (iii) mechanistic frameworks linking fabrication variables to material performance. These gaps have resulted in conflicting interpretations of performance drivers. For example, while some studies emphasize the role of fungal species—particularly white-rot basidiomycetes—in enhancing composite strength, others demonstrate that processing parameters such as density, moisture content, and post-treatment conditions exert a more dominant influence on mechanical behavior [1,3,10]. Thus, outcomes derived from specific experimental objectives often lack generalizability, underscoring the need for a more systematic and integrative understanding of MBC performance.

To address these limitations, this review adopts a fundamentally different perspective. Rather than reiterating existing knowledge, the study specifically aims to:

1. Synthesize quantitative data across studies to identify trends, variability, and inconsistencies;
2. Develop a PSP framework for MBC design and interpretation;
3. Critically evaluate methodological differences that contribute to variability in reported results;
4. Propose standardized testing protocols and reporting guidelines to improve reproducibility;
5. Assess technology readiness levels and scalability pathways for industrial adoption.

**Figure 1:**
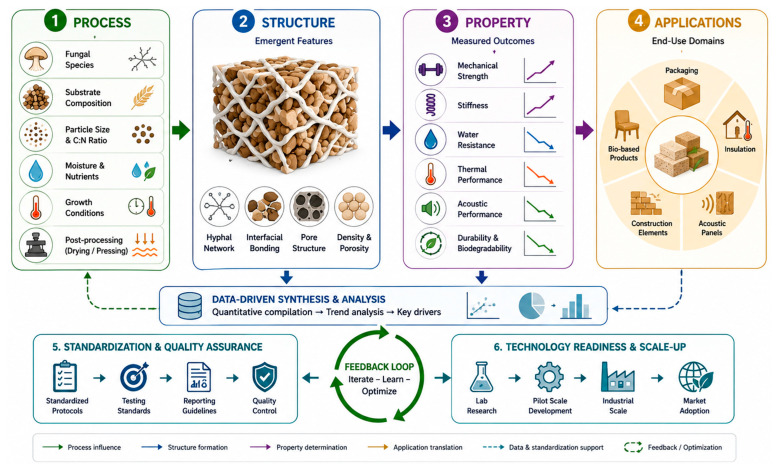
PSP framework for predictive design of lignocellulosic mycelium-bound composites.

A PSP-centered summary (Figure 1) itemizing the process variables, structures, properties, performance, and applications integrated with standardization and iterative optimization is shown below:

By transitioning from descriptive reporting to analytical integration, this work seeks to establish a foundation for predictive design and to facilitate the transition of MBCs from experimental biomaterials to reliable, application-ready composite systems. The schematic outlines the motivation for MBC development and positions the objective of this review in advancing standardized, scalable, and application-ready biomaterials through a PSP framework (Figure 1).

## 2. Review Methodology

This review employed a structured literature survey and quantitative synthesis approach to identify, evaluate, and integrate published studies on mycelium-bound composites (MBCs), with particular emphasis on the relationships among fungal species, substrate characteristics, processing conditions, post-treatment methods, and resulting material properties.

### 2.1. Literature Search Strategy and Referencing

Relevant literature was retrieved from major scientific databases, including Scopus, Web of Science Core Collection, ScienceDirect, MDPI, Google Scholar, and SpringerLink. The search was conducted between January and March 2026 and covered publications from 2010 to 2026, corresponding to the period during which research on mycelium-based materials experienced substantial growth and attention. The search utilized combinations of the following keywords and Boolean operators: “mycelium-bound composites”, “mycelium composites”, “mycelium-based materials”, “fungal biocomposites”, “biofabricated composites”, “lignocellulosic substrates”, “fungal biomaterials”, “mycelium insulation”, “mycelium packaging”, “fungal species”, “substrate engineering”, “densification”, “mechanical properties”, “compressive strength”, “thermal conductivity” and “biofabrication”. The citations and references were prepared using Mendeley Reference Manager.

### 2.2. Inclusion and Exclusion Criteria

Studies were included if they (1) reported experimental fabrication or characterization of mycelium-bound composites, (2) utilized fungal species to bind lignocellulosic or agricultural-residue substrates, (3) reported physical, mechanical, thermal, acoustic, biodegradation, or processing-related properties, (4) were published in peer-reviewed journals, and (5) were available in English.

Studies were excluded if they (1) focused exclusively on fungal biology, genetics, or cultivation without material fabrication, (2) addressed fungal-derived products unrelated to composite materials, (3) were conference abstracts, patents, editorials, book reviews, or non-peer-reviewed reports, (4) lacked sufficient methodological or property data for meaningful comparison, and (5) focused solely on leather-like mycelium materials without discussing composite formation.

### 2.3. Study Selection and Data Extraction

For each selected study, data were extracted on fungal species and strain (where available), substrate type and composition, carbon-to-nitrogen ratio, particle size and moisture content, growth conditions, post-processing methods (drying, pressing, heat treatment), density, compressive strength, elastic modulus, thermal conductivity, water absorption characteristics, and intended applications.

### 2.4. Quantitative Synthesis Approach

Rather than providing only a descriptive summary, the review compiled quantitative information from published studies to identify trends and sources of variability in MBC performance. Attention was given to relationships among processing variables, structural characteristics, and resulting material properties. The quantitative synthesis compiled reported values from 53 eligible studies to identify central performance trends and variability across fabrication strategies. Composite densities ranged from approximately 100 to 600 kg m^−3^, compressive strengths from 0.03 to 4.44 MPa, tensile strengths from 0.02 to 0.80 MPa, flexural strengths from 0.10 to 5.00 MPa, thermal conductivity from 0.03 to 0.09 W m^−1^ K^−1^, and water absorption from 30 to 400%, depending on fungal species, substrate composition, moisture content, and post-processing conditions. Rather than treating individual studies independently, these ranges were synthesized to identify consistent performance drivers, revealing that densification and moisture control explained substantially more variability than fungal species alone.

Data reported under different experimental conditions were critically interpreted within a Process–Structure–Property (PSP) framework, which was developed to explain how fungal species selection, substrate engineering, growth conditions, and post-processing collectively influence density, porosity, mechanical performance, and suitability for application.

Because experimental methodologies, specimen geometries, environmental conditions, and testing standards varied substantially across studies, the quantitative synthesis was intended to identify general trends and dominant performance drivers rather than establish universal predictive equations.

### 2.5. Limitations of the Literature Dataset

The reviewed literature exhibited substantial heterogeneity in substrate selection, fungal strains, incubation conditions, specimen preparation, and mechanical testing protocols. Consequently, direct comparison among studies should be interpreted with caution. Furthermore, incomplete reporting of strain-level information, environmental conditions, and post-processing parameters limited the extent of statistical normalization. These limitations further underscore the need for standardized testing and reporting protocols, which are among the central recommendations of this review.

### 2.6. Positioning of the Present Review Relative to Existing Reviews

Several review articles have summarized the development of MBCs, including fungal species selection, substrate utilization, fabrication methods, and emerging applications (Table 1). These studies have significantly advanced the understanding of mycelium-based materials and highlighted their potential as sustainable alternatives to conventional petroleum-based products. However, most existing reviews primarily adopt a descriptive approach, focusing on cataloging fabrication strategies, material properties, and application opportunities.

Despite these contributions, several important knowledge gaps remain. First, previous reviews generally provide limited quantitative synthesis of reported mechanical and physical property data across studies. Second, the relationships among processing variables, microstructural development, and resulting material properties are rarely integrated into a unified design framework. Third, standardization challenges associated with fabrication methods, testing protocols, and reporting practices have received relatively limited attention. Finally, few studies have evaluated TRLs or discussed pathways toward industrial-scale implementation.

To address these limitations, the present review adopts a PSP perspective that links fungal species selection, substrate engineering, growth conditions, and post-processing treatments to microstructural evolution and resulting material performance. In addition, this review provides a quantitative synthesis of properties reported in the literature, evaluates sources of variability, proposes standardization guidelines, and discusses technology readiness and scalability considerations. Table 1 summarizes the major differences between the present review and representative previously published reviews.

## 3. Quantitative Data Across Studies on Fungi Species Used in MBCs Production

Fungal species are critical determinants of mycelium-bound composite (MBC) performance, as they influence substrate colonization, hyphal architecture, enzymatic degradation, and interfacial bonding within lignocellulosic matrices [1,2,5]. White-rot fungi such as *Pleurotus ostreatus*, *Ganoderma lucidum*, and *Trametes versicolor* are the most extensively studied owing to their efficient lignocellulose utilization and binding capabilities [1,4]. However, reported mechanical properties vary widely across studies due to differences in substrates and processing conditions. Quantitative comparison is therefore essential to distinguish true species-specific effects from methodological variability and to guide strain selection for targeted MBC applications [3,5].

### 3.1. Species-Specific White-Rot Fungi and Their Influence on the Mechanical Performance of MBCs

The influence of species-specific white-rot fungi on the mechanical performance of MBCs has been widely investigated, primarily due to the central role of fungal hyphae in establishing the binding network within lignocellulosic substrates [12,13]. As summarized in Table 2, different fungal species exhibit varying capabilities in colonization efficiency, hyphal density, and interfacial bonding, which collectively contribute to differences in composite performance. However, a critical evaluation of the literature reveals that while species selection is important, its influence is often secondary to processing conditions and structural development of the intended products.

**Figure 2:**
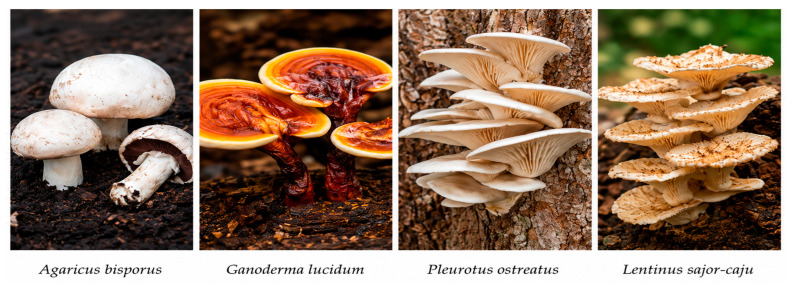
Basidiomycetes mushroom species commonly explored for mycelium-bound composites (MBCs) fabrication.

White-rot fungi, such as *Pleurotus ostreatus*, *Ganoderma lucidum*, and *Trametes versicolor,* are among the most utilized species (Figure 2) due to their ability to degrade lignin and facilitate substrate integration. These fungi produce extensive hyphal networks and secrete oxidative enzymes that modify lignocellulosic interfaces, enhancing adhesion between particles [20,21]. Oxidative enzymes such as laccases, manganese peroxidases, and lignin peroxidases catalyze selective oxidation and depolymerization of lignin phenolic structures while largely preserving cellulose microfibrils responsible for structural reinforcement [22,23]. This controlled biochemical modification increases substrate surface accessibility, exposes additional hydroxyl groups capable of hydrogen bonding, enhances hyphal penetration, and promotes stronger interfacial adhesion between fungal biomass and lignocellulosic particles [21,24,25]. Excessive enzymatic degradation, however, may weaken the substrate matrix, highlighting the importance of balancing biological modification with structural preservation [25].

For instance, *Ganoderma lucidum* has been reported to form dense, cohesive networks that enhance compressive strength, particularly when combined with densification processes (Table 2). Similarly, *Pleurotus ostreatus* demonstrates moderate baseline mechanical performance, which can be significantly enhanced through reinforcement and post-processing, achieving strengths approaching 2–3 MPa under optimized conditions.

Despite these species-specific differences, the variability in mechanical performance reported across studies remains substantial. As indicated in Table 2, compressive strength values range from 0.03 MPa to over 4 MPa for mixed white-rot systems, highlighting the inherent inconsistency in current fabrication approaches. This variability is consistent with broader findings in the literature, where identical fungal species have produced composites with markedly different properties depending on substrate type, moisture content, and incubation conditions [1,5]. Such observations suggest that species alone does not dictate performance but rather interacts with a complex set of process variables.

A key factor mediating species influence is the development of “hyphal network architecture.” Fungal species differ in hyphal thickness, branching patterns, and growth rates, which affect network connectivity and load transfer within the composite [6]. For example, rapidly colonizing species, such as *Trametes versicolor* can achieve uniform substrate coverage, contributing to efficient stress distribution and favorable strength-to-weight ratios (Table 2). However, these advantages may be offset if growth conditions are not optimized, leading to incomplete binding or excessive porosity [10,17].

Another important consideration is the enzymatic activity of white-rot fungi. Of particular interest is the fungi’s ability to selectively modify lignin without extensively degrading cellulose. Controlled lignin modification enhances interfacial bonding while preserving the structural integrity of cellulose fibers, which are critical for mechanical strength [21,26]. Nevertheless, excessive enzymatic degradation can weaken the substrate matrix, underscoring the need for balanced biological activity during fabrication.

Importantly, the data presented in Table 2 reinforce the growing consensus that processing conditions, particularly densification, exert a dominant influence on mechanical performance. Studies have consistently shown that the pressing and drying operations in MBC production significantly increase composite density, reduce porosity, and enhance inter-particle contact, leading to substantial improvements in compressive strength [1,3]. In many cases, these processing effects outweigh species-specific differences, explaining why reinforced or densified *Pleurotus*-based composites can outperform non-pressed systems produced with other fungi (Table 2).

From a materials design perspective, this indicates that fungal species should be considered part of an integrated system rather than as the primary determinant of performance. The role of the fungus is to establish a functional binding network, while the final mechanical properties are governed by the combined effects of substrate composition, growth conditions, and post-processing treatments. This interpretation aligns with the process–structure–property framework, where species selection influences microstructural development but does not solely define macroscopic behavior.

### 3.2. Implications for Comparative Performance and Material Optimization

The variability highlighted in Table 3 has important implications for both research and application. First, it underscores the need for standardized testing and reporting protocols, as differences in sample preparation and measurement conditions can obscure true species-specific effects. Second, it suggests that future research should focus on systematic comparisons under controlled conditions, where species, substrate, and processing variables are independently evaluated.

Moreover, the ability of certain species–process combinations to achieve compressive strengths exceeding 2 MPa (Table 3) indicates that MBCs can approach the performance of low-density synthetic materials, particularly when optimized through densification and reinforcement strategies. However, achieving consistent performance at this level remains a challenge due to biological variability and sensitivity to environmental conditions.

Overall, the evidence supports a shift in research emphasis from identifying “optimal” fungal species to developing integrated design strategies that combine species selection with controlled processing and substrate engineering. By doing so, it becomes possible to harness the unique capabilities of white-rot fungi while minimizing variability and improving reproducibility in MBC fabrication.

### 3.3. MBCs and Conventional Adhesive-Bonded Materials

White-rot fungi are widely employed in the fabrication of mycelium-bound composites (MBCs) because of their ability to colonize lignocellulosic substrates and form interconnected hyphal networks that act as natural binders. Species differ in hyphal morphology, enzymatic activity, and colonization behavior, influencing microstructural features such as hyphal density, pore distribution, and interfacial adhesion. For example, *Pleurotus ostreatus* and *Ganoderma lucidum* develop dense, interconnected mycelial networks (Figure 2) that enhance particle bonding and structural cohesion [1,17], while *Trametes versicolor* exhibits rapid colonization and favorable strength-to-weight performance through uniform network formation [18].

Despite these biological differences, increasing evidence suggests that fungal species alone do not govern composite mechanical performance [16,27]. Instead, performance results from the interaction between species characteristics and processing variables, including substrate composition, moisture content, and post-processing treatments such as pressing and drying [9,19]. Accordingly, identical fungal species can produce composites with markedly different mechanical properties under different processing conditions, consistent with the broad range of compressive strengths reported in the literature (Table 3).

A critical factor moderating species influence is the density enhancement of the products during fabrication. While fungal growth establishes the initial binding network, densification processes—particularly mechanical pressing—significantly enhance load-bearing capacity by reducing porosity and increasing inter-particle contact. This explains why densified MBCs exhibit substantially higher compressive strength than their non-pressed counterparts, regardless of species selection (Table 2). To facilitate a more meaningful comparison between MBCs and conventional adhesive-bonded composites, the discussion has been expanded to include density-normalized performance. While resin-bonded materials such as particleboard and MDF exhibit substantially higher absolute compressive strengths, they also possess considerably greater densities. When mechanical performance is evaluated relative to density, densified MBCs exhibit a competitive specific strength compared with other lightweight insulating materials, particularly expanded polystyrene (EPS) and hempcrete (Table 3). This normalization highlights that MBCs are better evaluated within the context of lightweight bio-based materials rather than as direct substitutes for high-density structural panels. In this context, fungal species play a facilitative rather than dominant role, enabling network formation that is subsequently optimized through engineering processes.

**Table 3:** Comparison of mycelium-bound composites (MBCs) with conventional lightweight and engineered composite materials.

* Material	Binder System	Density (kg/m^3^)	Compressive Strength (MPa)	Specific Compressive Strength (MPa·m^3^/kg × 10^−3^)	Tensile Strength (MPa)	Flexural Strength (MPa)	Conductivity (W/m·K)	Water Absorption (%)	Refs.
MBC (non-pressed)	Biological hyphal network	100–400	0.03–2.5	0.3–6.3	0.02–0.30	0.10–1.20	0.03–0.08	100–400	[1,17,19]
MBC (pressed)	Biomechanical	200–600	1.0–4.4	5.0–10.0	0.10–0.80	0.50–5.00	0.04–0.09	30–150	[14,17,19]
EPS	Synthetic polymer	10–40	0.07–0.40	7.0–10.0	0.10–0.70	0.15–1.00	0.030–0.040	<5	[28,29,30]
Hempcrete	Lime-based mineral	200–400	0.20–0.50	0.5–2.5	0.02–0.10	0.10–0.50	0.05–0.12	50–200	[31]
Particleboard	Urea-formaldehyde	600–800	5–25	8.3–41.7	5–15	10–30	0.10–0.15	5–40	[32,33]
MDF	Synthetic resin	600–900	10–40	11.1–66.7	10–30	20–50	0.10–0.18	5–30	[34,35]
Cork board	Natural suberin	100–250	0.20–2.0	2.0–8.0	0.20–1.0	0.50–3.0	0.035–0.065	10–50	[30]
Wood fiber insulation board	Lignin/self-bonding	110–270	0.05–1.0	0.4–4.0	0.05–0.50	0.10–2.0	0.037–0.055	20–120	[32,36]

* Values represent ranges reported in the literature and should be interpreted as indicative rather than directly comparable because of differences in specimen geometry, testing protocols, moisture conditioning, and fabrication methods.

#### 3.3.1. Comparison with Conventional Adhesive-Based Composites

**Figure 3:**
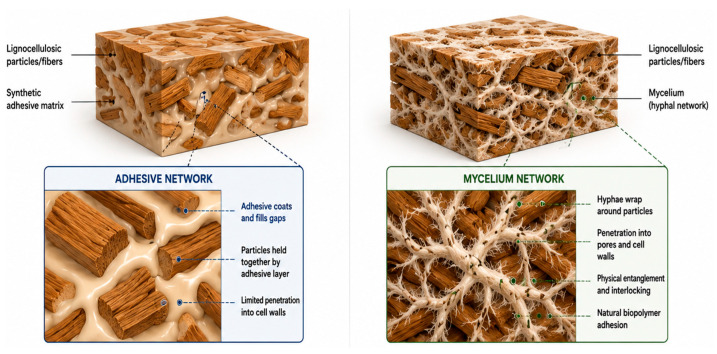
Schematic comparison of conventional adhesive-based composites and mycelium-bound composites. Conventional composites rely on synthetic adhesives that coat particle surfaces and bridge voids with limited penetration. In contrast, mycelium-bound composites utilize fungal hyphal networks that infiltrate, entangle, and interlock lignocellulosic substrates, resulting in a natural, 3D binding architecture.

The role of white-rot fungi in MBCs contrasts fundamentally with that of synthetic adhesives used in conventional composite materials. In adhesive-based systems, such as particleboard and medium-density fiberboard (MDF), binding is achieved through thermosetting resins (e.g., urea-formaldehyde or phenol-formaldehyde) that form continuous polymer matrices with strong covalent crosslinking (Figure 3) [31]. This results in highly uniform and predictable mechanical performance, with compressive strengths typically exceeding 5 MPa and reaching up to 40 MPa in engineered panels.

In contrast, MBCs rely on biologically mediated binding mechanisms, including hyphal entanglement, extracellular polymer secretion, and partial enzymatic modification of lignin and polysaccharides [37,38]. These interactions produce a heterogeneous and discontinuous network structure, which inherently limits mechanical strength and introduces variability. As summarized in Table 4, the continuity of the structure and bonding chemistry of synthetic adhesives provides a significant advantage in terms of mechanical performance and reproducibility.

Nevertheless, MBCs demonstrate competitive performance in specific application domains. For example, their compressive strength overlaps with that of expanded polystyrene (EPS) and other lightweight insulating materials, making them suitable for non-structural applications such as packaging and thermal insulation (Table 3). Moreover, unlike synthetic adhesives, fungal binding systems are biodegradable and derived from renewable resources, offering substantial environmental benefits [2,35].

Another key distinction lies in process sensitivity. While well-controlled chemical curing reactions govern adhesive-based composites, MBCs are influenced by biological variability, which includes environmental conditions, nutrient availability, and microbial interactions. This contributes to the higher variability observed in MBC performance (Table 3) and underscores the need for standardized fabrication and testing protocols.

#### 3.3.2. Integrating Species Effects Within a PSP Framework

To reconcile the apparent inconsistencies in species-specific performance, it is essential to situate fungal selection within a broader process–structure–property (PSP) framework. Within this framework, fungal species primarily influence: (i) growth kinetics and colonization efficiency, (ii) hyphal morphology and network connectivity, and (iii) enzymatic modification of substrate interfaces.

However, the translation of these biological characteristics into mechanical performance is mediated by: (i) substrate composition and particle size, (ii) moisture content and incubation conditions, and (iii) post-processing parameters such as pressing pressure and drying rate.

#### 3.3.3. Implications for Material Design and Research Direction

A comparative analysis of the reported findings (Table 2 and Table 3) highlights a fundamental trade-off between mechanical performance and sustainability. While synthetic adhesives provide superior strength and consistency, they are associated with environmental and health concerns, including formaldehyde emissions (which are considered more significant in contemporary manufacturing) and non-biodegradability. In contrast, MBCs present a renewable, low-carbon alternative, albeit one that requires further optimization to achieve consistent, scalable performance.

Based on the findings, future research, among other possibilities, should therefore focus on:

(i) Systematic evaluation of species–substrate compatibility: In this context, systematic compatibility assessment refers to the structured evaluation of fungal species against agricultural residues based on lignocellulosic composition, carbon-to-nitrogen ratio, particle morphology, moisture retention, colonization rate, and enzymatic compatibility. Such assessment enables the identification of species–substrate combinations that maximize hyphal penetration, interfacial bonding, and composite density while minimizing biological variability. Agricultural residues such as wheat straw, corn stover, hemp hurd, rice husk, and sawdust differ substantially in chemical composition and physical architecture; therefore, compatibility assessment provides a rational basis for selecting feedstocks capable of producing reproducible composite properties.
(ii) Quantitative correlation of processing parameters with mechanical outcomes.
(iii) Development of standardized testing protocols, and
(iv) Integration of hybrid reinforcement strategies to bridge performance gaps. By adopting a systems-level approach, it becomes possible to harness the unique advantages of white-rot fungi while mitigating their limitations. This will enable the transition of MBCs from experimental biomaterials to reliable, application-ready composite systems.

## 4. Substrate Selection and Process–Structure–Property Relationships in MBCs

### 4.1. Properties of Lignocellulosic Substrates

The selection of lignocellulosic substrates is a fundamental determinant of MBC performance, as substrate properties govern both fungal colonization and the resulting material structure [14,16]. Unlike synthetic composites, where matrix properties dominate, MBCs rely heavily on the intrinsic physicochemical characteristics of the substrate, which define the initial scaffold for hyphal binding and structural development [39].

#### 4.1.1. Carbo-to-Nitrogen (C:N) Ratio of the Substrate

The substrate’s C:N ratio is a key factor of fungal mycelium metabolic efficiency and composite morphology. The growth of mycelium in MBC systems is typically optimized at C:N ratios ranging from around 20:1 to 30:1, depending on the species and substrate types [40]. This range supports hyphal branching, enzyme production, and cell wall synthesis without excessive softening of the biomass [38]. Nitrogen limitation within this range encourages fungi to invest in exploratory growth and network formation, which consequently enhances particle binding [41,42].

Conversely, excess nitrogen in the substrates has been shown to cause rapid but poorly structured mycelial growth, which loosens matrices and compromises the mechanical integrity of the composite [43,44]. High nitrogen levels can also restrain ligninolytic enzyme expression, reducing substrate modification and deteriorating interparticle cohesion [10].

Recent experimental work has demonstrated that nitrogen-rich substrates often produce composites with lower compressive strength and increased brittleness, underscoring the importance of maintaining an optimal C:N balance [38,45]. Table 5 shows various C:N ratios of some common substrates explored so far in MBC research. Substrates such as straw and sawdust often require supplementation (e.g., bran, DDGS) to achieve balanced nutrient availability. Studies have shown that improper C:N ratios can result in heterogeneous colonization and weak inter-particle bonding, directly affecting composite strength [21,26].

In summary, the high C:N ratio (carbon-rich substrates) promotes structural integrity but may limit fungal growth due to nitrogen deficiency, whereas the low C:N ratio (nitrogen-rich substrates) enhances growth rate but can lead to excessive biomass formation and reduced mechanical stability.

#### 4.1.2. Particle Size and Physical Structure

The substrate particle size distribution plays a dual role, influencing both mycelial binding efficiency and gas exchange within the composite. Smaller particles increase surface area and contact points for hyphal attachment, often resulting in denser and mechanically stronger composites [21]. It was demonstrated that reduced particle size significantly improved compressive strength due to tighter mycelial encapsulation and reduced void volume [34]. However, excessively fine particles can hinder oxygen diffusion and restrict hyphal respiration, leading to incomplete colonization or weak interfacial bonding [28]. In contrast, larger particles enhance porosity and aeration but may reduce interparticle contact, resulting in lower density and mechanical performance [49]. As a result, recent studies advocate for graded or mixed particle size distributions, which balance gas permeability with effective mycelial binding [45,46]. Experimental studies indicate that intermediate particle sizes often produce optimal results by balancing mechanical interlocking and biological accessibility [1].

A quantitative survey of the reviewed literature indicates that substrate particle sizes investigated for MBC production generally fall into three categories: fine (<0.5 mm), intermediate (0.5–2.0 mm), and coarse (>2.0 mm). Fine particles typically produce denser composites with improved compressive strength because of greater packing density and enhanced hyphal encapsulation, although excessive fines may reduce pore connectivity and restrict oxygen diffusion during colonization [1,48]. Conversely, coarse particles (>2 mm) improve aeration and gas exchange but generally increase internal void volume, resulting in lower density and reduced mechanical strength [28]. Intermediate particle sizes (0.5–2.0 mm), or graded blends combining fine and coarse fractions, consistently provide the best balance between biological accessibility, gas permeability, and structural reinforcement and therefore represent the most frequently recommended particle-size range for MBC fabrication [1,45,50].

#### 4.1.3. Moisture Content and Water Availability

Moisture content is another important characteristic that influences both fungal physiology and composite structure. Adequate water availability promotes food transport, enzymatic activity, and hyphal expansion, whereas insufficient moisture inhibits metabolic processes and leads to incomplete colonization. Several studies have found that the ideal starting moisture level for several lignocellulosic substrates used in MBCs is between 60 and 70% (wet basis) [51,52]. Results show that increasing substrate moisture content to 65–70% resulted in composites with higher density, homogeneity, and compressive strength, which they attributed to increased hyphal penetration and fewer internal voids [51].

However, excessive dampness can be equally problematic. Substrates’ moisture contents that exceed the ideal range may cause oxygen limitation, the formation of anaerobic microenvironments, and increased susceptibility to bacterial contamination, all of which have a detrimental influence on composite quality [10,52]. Recent research has highlighted the connection between moisture content and substrate porosity, implying that ideal moisture levels depend both on water content and on particle geometry and packing density [47]. These findings highlight the importance of optimizing substrate-specific moisture rather than relying on universal values.

### 4.2. Extrinsic MBCs’ Substrate Properties

While intrinsic substrate properties define the initial framework, extrinsic processing conditions ultimately determine the final material performance. These parameters are often underreported yet exert dominant control over mechanical behavior.

#### 4.2.1. Mixing and Inoculation Strategies

Beyond intrinsic substrate features, substrate handling and preparation processes are other extrinsic factors that influence the composite’s uniformity and structural integrity [16,51]. Substrate mixing strategy plays a critical role in achieving uniform inoculum distribution, moisture homogeneity, and consistent mycelial colonization. At the laboratory scale, manual batch mixing is the most widely used approach because it enables thorough incorporation of fungal spawn into chopped lignocellulosic substrates before molding [1,2]. For larger-scale production, rotary drum mixers and ribbon (horizontal paddle) mixers provide more uniform blending while minimizing particle segregation and spawn damage [7,10]. In solid-state fermentation systems, tray-based cultivation with periodic manual turning or pile turning is employed to improve aeration, redistribute moisture, and reduce localized overheating and contamination during colonization [52]. More advanced pilot-scale systems utilize moving-floor or conveyor-based solid-state bioreactors, which continuously mix and aerate substrates to improve process uniformity and scalability [53]. Collectively, these mixing strategies promote homogeneous hyphal network development, reduce density gradients, and improve the reproducibility of mechanical properties in mycelium-bound composites.

Distributing fungal inoculum evenly across the substrate before incubation culminated in denser composites with fewer voids than surface or multilayer inoculation approaches [52]. Homogeneous inoculation enhances hyphal–substrate contact while reducing localized nutrient depletion, resulting in more uniform mycelial networks and improved mechanical performance. In contrast, unequal inoculation can result in preferential growth zones, internal defects, and anisotropic mechanical behavior [47,54].

These findings highlight that substrate characteristics cannot be considered independent of processing methods. Instead, substrate chemistry, particle geometry, and mixing strategies act synergistically to determine final composite performance.

#### 4.2.2. Implications for Substrate Design in MBCs

Recent literature collectively emphasizes that substrate selection for MBCs must be tailored to individual applications and empirically refined [1,5,15,16,55]. For load-bearing or semi-structural applications, substrates must possess a balanced lignocellulosic composition, moderate particle size, controlled moisture content, and an optimal carbon-to-nitrogen ratio to ensure adequate strength and dimensional stability [17]. In contrast, lightweight insulation materials can accommodate greater porosity and reduced density, permitting coarser substrates and wider moisture variations [31].

Current research increasingly views substrates as active design components rather than merely fillers, with their chemical and physical properties adjustable to influence fungal growth and material performance [39]. This paradigm change establishes substrate engineering and fungal species selection as fundamental elements of MBC material design. The lignocellulosic composition, C:N ratio, moisture content, and particle-size distribution collectively affect enzymatic activity, hyphal penetration, and matrix consolidation [1,5,11]. Research findings also underscore that optimal composite performance arises from a careful balance of these factors, supported by appropriate mixing and inoculation strategies [56,57,58].

As MBC research progresses toward scalable, application-specific implementation, thorough substrate optimization will remain fundamental to reliable, reproducible composite manufacturing.

## 5. Post-Treatment: Drying, Pressing, and Functional Transformation of MBCs

Following incubation, biological activity must be terminated, and the composite stabilized to achieve reproducible material performance. Post-treatment—primarily controlled drying and mechanical pressing—is therefore not merely a preservation step but a critical transformation stage that governs the transition from a biologically assembled structure to an engineering material.

### 5.1. Drying-Induced Structural Stabilization

Drying removes free and bound water, arrests fungal metabolism, and fixes the microstructure formed during growth. However, the drying rate and temperature strongly influence internal stress development and structural integrity. Rapid drying at elevated temperatures can create steep moisture gradients between the surface and core, generating tensile stresses that lead to cracking, warping, or collapse of the hyphal network [1,5]. Conversely, insufficient drying leaves residual moisture that promotes microbial degradation and dimensional instability over time [2].

Experimental studies indicate that moderate drying conditions (≈40–70 °C) provide a balance between structural preservation and efficient moisture removal [4]. Controlled drying conditions significantly influence porosity and stiffness, with excessive thermal exposure leading to embrittlement and microstructural damage [3]. These findings underscore that drying is not a neutral step but a structure-defining process that directly affects mechanical outcomes.

### 5.2. Pressing and Densification: Dominant Drivers of Mechanical Performance

**Figure 4:**
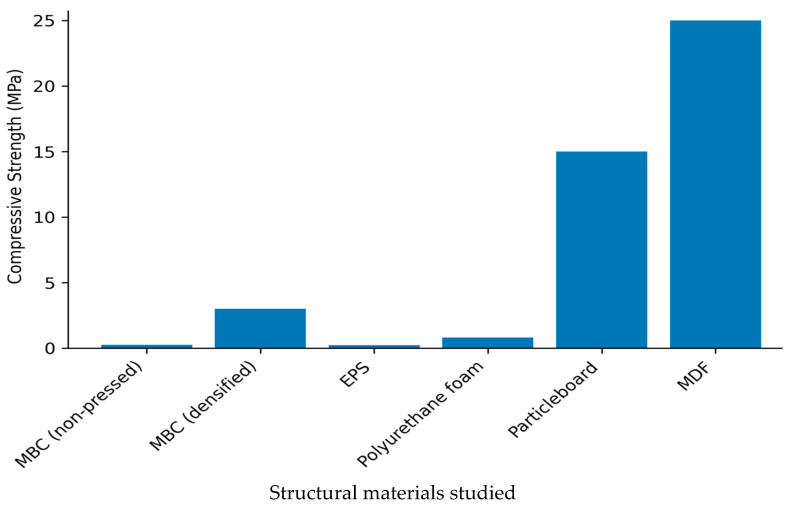
Comparison of compressive strength ranges for mycelium-bound composites (MBCs) and conventional materials. Non-pressed MBCs exhibit low strength (<0.5 MPa), while densified MBCs can reach ~3–4 MPa. These values overlap with those of expanded polystyrene (EPS) and low-density polyurethane foams but remain significantly lower than those of resin-bonded composites such as particleboard and medium-density fiberboard (MDF), which exhibit strengths above 5 MPa.

Mechanical pressing represents the most influential post-treatment parameter for enhancing MBC performance. Pressing increases density by reducing pore volume, improving interparticle contact, and promoting load-transfer pathways. Quantitatively, studies have shown that compressive strength can increase by an order of magnitude after densification, with values rising from <0.5 MPa in unpressed samples to >2–4 MPa in pressed systems (Figure 4) [1,4].

Beyond densification, pressing alters the functional roles of both the substrate and the mycelium: (i) The *substrate* (lignocellulosic phase) acts as the primary load-bearing skeleton. Under pressure, particles rearrange, collapse, and interlock, forming a continuous stress-transfer network analogous to fiber reinforcement in conventional composites [1]. (ii) The *mycelium* (biological phase) functions as a binding and interfacial modifier, rather than the primary load-bearing component. During pressing, hyphal networks are compressed and redistributed, enhancing adhesion through increased contact area and localized bonding, but their structural contribution becomes secondary to interactions with the densified substrate [30,59].

This distinction is critical and often overlooked. While hyphal morphology and growth influence initial cohesion, mechanical performance in heat-pressed systems is predominantly governed by substrate densification rather than intrinsic fungal properties [1,6].

#### 5.2.1. Thermo-Mechanical Effects in Heat-Pressed Systems

In heat-assisted pressing, additional physicochemical transformations occur. Elevated temperatures can soften lignin and hemicellulose, enabling thermoplastic deformation and partial re-bonding of lignocellulosic components, which further enhances mechanical integrity [36]. Simultaneously, fungal biomass undergoes thermal inactivation and structural consolidation. Pressing temperature and pressure jointly influence density, stiffness, and water resistance, with higher pressures producing more uniform and mechanically stable composites [1]. Similarly, pressing at ~1–2 MPa significantly improves compressive strength and reduces water absorption by minimizing pore connectivity [60]. However, excessive pressure or temperature may damage the hyphal network or induce brittle behavior, indicating the need for optimized processing windows rather than maximum densification.

#### 5.2.2. Integration of Growth and Post-Processing Effects

A key limitation in the current literature is the tendency to attribute mechanical performance solely to hyphal properties, without adequately accounting for post-processing effects [39]. MBC performance emerges from a two-stage process: (i) biological stage (growth)—establishes a continuous binding network and initial structural cohesion; (ii) engineering stage (post-processing)—determines density, porosity, and load-bearing capacity. This explains why materials produced with similar fungal species can exhibit vastly different mechanical properties depending on pressing and drying conditions. It also clarifies why only densified (heat-pressed) MBCs demonstrate potential for semi-structural or load-bearing applications, whereas non-pressed materials remain suitable primarily for insulation or packaging [18,46].

#### 5.2.3. Implications for Application and Design

The transformation induced by drying and pressing enables MBCs to behave more like conventional engineering materials, with improved dimensional stability, reduced water absorption, and more predictable mechanical properties. However, unlike synthetic composites, these properties remain highly sensitive to processing conditions.

For practical applications, non-pressed MBCs are suitable for low-density applications (e.g., packaging, insulation), and pressed MBCs show potential for semi-structural uses, provided density and uniformity are controlled. The future work should therefore prioritize: (a) quantitative relationships between pressure–density–strength; (b) standardized reporting of post-processing parameters; and (c) mechanistic understanding of substrate–mycelium interactions under compression.

## 6. Methodological Variability in MBC Synthesis: Sources, Consequences, and Pathways to Standardization

**Figure 5:**
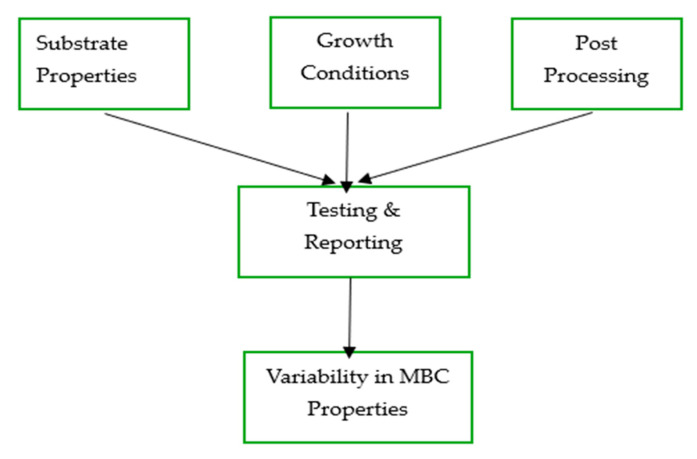
Schematic representation of major sources of variability in mycelium-bound composite (MBC) synthesis. Intrinsic factors such as substrate properties and growth conditions, together with extrinsic processing parameters, converge to influence testing and reporting outcomes, ultimately leading to variability in measured mechanical and physical properties.

The rapid expansion of MBC research has been accompanied by substantial variability in reported mechanical, physical, and durability properties. This variability is not solely attributable to biological differences. Still, it arises largely from methodological inconsistencies across studies, including differences in substrate preparation, inoculation protocols, growth conditions, post-processing, and testing procedures (Figure 5). As a result, direct comparison between studies is often unreliable, limiting the development of predictive design frameworks and hindering industrial translation. The methodological factors contributing to variability in MBC synthesis and performance, highlighting both common practices and key disparities, are critically evaluated.

### 6.1. Variability in Substrate Preparation and Composition

Substrate formulation is one of the most significant sources of variability. Studies differ widely in: (i) type of lignocellulosic feedstock (e.g., straw, sawdust, agricultural residues, distiller’s grain with solubles); (ii) particle size distribution; (iii) pre-treatment methods (sterilization vs. pasteurization); and (iv) nutrient supplementation. Table 6 summarizes the variability in substrate preparation across studies.

For example, studies demonstrated that particle size and substrate type significantly influence density and compressive strength, with finer particles producing denser but less aerated structures [5]. Similarly, substrate composition affects moisture retention and fungal colonization, leading to variability in mechanical performance [1].

### 6.2. Inoculation and Growth Conditions

Fungal inoculation methods and incubation conditions vary significantly across studies, including spawn type (grain, liquid culture), inoculum loading (typically 5–20%), temperature (20–30 °C), relative humidity (>70%), and incubation time (5–21 days). These parameters influence hyphal growth rate, network formation, and substrate colonization. For instance, incubation time strongly affects internal bonding, with insufficient colonization leading to weak composites [2]. However, excessive growth can result in uneven density distribution and structural heterogeneity. Table 7 highlights the growth conditions that act as sources of variability in MBCs.

### 6.3. Post-Processing: Drying, Pressing, and Densification

Post-processing is the dominant factor controlling final mechanical performance, yet it is inconsistently applied and often poorly reported. Key variations include drying temperature (25–80 °C), drying duration (hours to days), pressing pressure (0–2 MPa), and use of heat-assisted pressing [1].

It has also been demonstrated that compressive strength correlates strongly with density, which is primarily controlled by pressing [1]. Similarly, drying conditions have also been reported to influence stiffness and porosity significantly [3]. Despite these findings, many studies do not report pressing parameters in detail, making reproducibility difficult. Table 8 summarizes the post-processing variability and its effect on the properties of the resultant MBCs.

### 6.4. Mechanical Testing and Reporting Inconsistencies

A major limitation in the literature is the lack of standardized testing protocols (Table 9). Studies differ in sample geometry and size, loading rate, moisture conditioning, and reporting metrics (e.g., peak stress vs. modulus). It was reported that compressive strength values cannot be directly compared due to differences in testing conditions [1]. This leads to apparent discrepancies in which similar materials exhibit widely different reported properties.

The combined effect of these methodological differences is a high degree of variability in reported properties, with compressive strength ranging from <0.1 MPa to >4 MPa across studies [1,3]. This variability complicates cross-study comparison, identification of performance drivers and development of predictive models. Importantly, many discrepancies attributed to biological factors (e.g., fungal species) are in fact the result of uncontrolled process variables, particularly density and moisture content.

### 6.5. Toward Standardization and Best Practices

To address these challenges, the field must adopt standardized methodologies, including:

- Substrate Characterization: Particle size distribution and C:N ratio.
- Controlled Growth Conditions: Temperature, humidity, incubation time.
- Defined Post-processing: Pressing pressure, drying temperature.
- Standard Testing Protocols: Sample size, loading rate, conditioning.

Adopting such standards will enable: (i) reliable comparison across studies, (ii) identification of true performance drivers, and (iii) acceleration of industrial adoption.

### 6.6. Literature-Based Validation of the Process–Structure–Property (PSP) Framework

The proposed Process–Structure–Property (PSP) framework postulates that processing variables—including fungal species selection, substrate characteristics, incubation conditions, and post-processing treatments—influence the microstructural architecture of MBCs, which subsequently governs their physical and mechanical properties. To assess the validity of this framework, representative studies from the literature were examined to determine whether observed property changes can be explained through PSP relationships.

#### 6.6.1. Case Study I: Densification as a Process Driver

Mechanical densification represents one of the clearest examples supporting the PSP framework. Several studies reported substantial increases in compressive strength following pressing operations. It has been demonstrated that heat-pressed mycelium composites exhibited significantly greater density and stiffness than unpressed counterparts [1]. Similarly, findings reported that compressive strength increased from values below 0.5 MPa in loosely consolidated systems to values exceeding 2 MPa following densification [10,61]. Further investigations posited and ascertained that fiber-reinforced and densified composites achieved compressive strengths approaching 2.9 MPa [4].

Within the PSP framework, pressing pressure functions as the primary process variable. Densification reduces pore volume, increases particle-particle contact, and promotes a more continuous load-transfer network within the composite structure. These structural modifications subsequently translate into improved compressive strength and elastic modulus. The consistency of these observations across multiple studies suggests that densification exerts a predictable and reproducible influence on MBC performance regardless of fungal species or substrate selection.

#### 6.6.2. Case Study II: Substrate Engineering and Structural Development

The influence of substrate properties further supports the PSP framework. Investigations revealed that particle size distribution significantly influences composite density and porosity [3,62]. Smaller substrate particles increase packing efficiency and provide greater surface area for hyphal attachment, resulting in denser microstructures and improved mechanical performance. Conversely, excessively coarse particles increase void content and reduce structural cohesion [60].

Similarly, substrates possessing balanced carbon-to-nitrogen ratios (approximately 20:1–30:1) promote uniform colonization and network formation, whereas excessively high or low C:N ratios can impair either fungal growth or structural integrity [39,40,41,63]. These findings demonstrate that substrate characteristics influence microstructural development by altering hyphal penetration, pore distribution, and interfacial bonding, ultimately determining the material properties.

The observed relationships are therefore consistent with the PSP hypothesis that substrate selection acts primarily through structural modification rather than direct property enhancement.

#### 6.6.3. Case Study III: Species Effects Versus Processing Effects

A major objective of this review was to determine whether fungal species or processing conditions exert the greater influence on mechanical performance. The compiled literature suggests that species-specific effects exist but are often secondary to structural changes induced by processing. For example, Ganoderma lucidum has been reported to produce dense hyphal networks associated with enhanced compressive strength [16], while Trametes versicolor exhibits rapid colonization and favorable strength-to-weight performance [64]. However, composites fabricated from the same fungal species frequently exhibit order-of-magnitude differences in compressive strength when different substrates, moisture conditions, or post-processing methods are employed [39,51,65]. Pleurotus ostreatus composites provide a representative example. Depending on processing conditions, reported compressive strengths range from approximately 0.5 MPa to nearly 3 MPa [60]. Such variability cannot be explained by species selection alone and instead reflects changes in density, porosity, and interfacial bonding resulting from substrate engineering and densification processes.

These observations support the PSP framework’s central premise that fungal species influence structural formation, whereas final performance emerges from the interaction between biological and engineering variables.

#### 6.6.4. Comparative Evidence Supporting PSP Relationships

Table 10 summarizes representative examples from the literature that demonstrate agreement between PSP predictions and experimentally observed outcomes.

#### 6.6.5. Limitations and Future Validation

Although the literature consistently supports PSP relationships, several limitations remain. Experimental methodologies, specimen dimensions, environmental conditioning, fungal strains, and testing standards vary considerably among studies. Consequently, the current validation should be viewed as trend-based rather than fully predictive. Furthermore, long-term durability, biodegradation, aging effects, and variables in industrial-scale cultivation remain insufficiently represented in existing datasets.

Future work should focus on generating standardized experimental databases that enable quantitative regression modeling, machine-learning approaches, and multivariate analysis. Such efforts would facilitate development of predictive equations capable of estimating material properties from process variables and further strengthen the PSP framework as a design tool.

Overall, the literature demonstrates that modifications in processing variables consistently generate predictable structural changes that are reflected in material performance. This evidence supports the PSP framework as a scientifically grounded approach for interpreting and guiding the design of mycelium-bound composites.

## 7. Technology Readiness Levels (TRLs) and Scalability Pathways for Industrial Adoption of MBCs

The transition of MBCs from laboratory-scale materials to industrial products remains constrained by technological, economic, and process-integration challenges [1,39]. While significant advances have been made in understanding fungal growth and material fabrication, the field is still largely positioned within low-to-mid TRLs, with only a limited number of applications approaching commercialization [66].

TRL provides a structured framework for evaluating the maturity of MBC systems, ranging from TRL 1 (concept validation) to TRL 9 (full-scale deployment). Most MBC research currently resides between TRL 3 and TRL 5, where laboratory validation and early prototyping dominate [1,2]. Commercial applications—primarily in packaging and insulation—have reached higher readiness levels (TRL 7–9), but these represent relatively low-performance use cases compared to structural materials.

A major barrier to advancing TRL lies in the biological nature of MBC fabrication, which introduces variability not typically encountered in synthetic material systems. Unlike polymer composites that rely on controlled chemical reactions, MBC production depends on living organisms whose growth is sensitive to environmental conditions such as temperature, humidity, and nutrient availability [7]. This sensitivity complicates scale-up, as maintaining uniform growth conditions across large volumes is technically challenging.

### 7.1. Scalability Constraints in MBC Production

Despite promising laboratory results, several bottlenecks hinder the large-scale manufacturing of MBCs. These challenges can be broadly categorized into biological, engineering, and logistical constraints.

#### 7.1.1. Biological Constraints

MBC production relies on living systems, introducing variability that is absent in conventional composite manufacturing. Factors such as contamination risk, inconsistent colonization, and sensitivity to environmental conditions can lead to batch-to-batch variability [40]. Additionally, growth cycles (typically several days to weeks) are significantly longer than synthetic manufacturing processes, limiting throughput.

#### 7.1.2. Process and Engineering Constraints

Scaling MBC production requires precise control over temperature and humidity, aeration and oxygen diffusion, and moisture gradients within bulk substrates. In large-scale systems, achieving uniform colonization becomes increasingly difficult due to mass transfer limitations, particularly in dense or thick geometries [3]. Furthermore, post-processing steps such as pressing and drying must be carefully controlled to avoid structural defects, as previously discussed.

#### 7.1.3. Material Variability and Feedstock Supply

Unlike petrochemical feedstocks, lignocellulosic substrates exhibit inherent variability in composition and moisture content, which affects reproducibility. Seasonal and geographical variations in agricultural residues further complicate standardization [21].

### 7.2. Comparison with Conventional Composite Manufacturing

The scalability challenges of MBCs can be better understood by comparison with conventional composite systems. Synthetic composites benefit from: (i) continuous production processes, (ii) controlled raw material composition, and (iii) rapid curing times.

In contrast, MBC production is inherently batch-based and biologically driven, resulting in longer processing times and higher variability. For example, particleboard manufacturing can be completed within minutes under controlled conditions, whereas MBC growth cycles may require several days [35].

However, MBCs offer advantages in: (i) lower energy consumption, (ii) reduced carbon footprint, and (iii) elimination of synthetic adhesives.

Life cycle assessments have shown that mycelium-based materials can significantly reduce environmental impact compared to petroleum-based foams, particularly in terms of greenhouse gas emissions and end-of-life disposal [67].

### 7.3. Emerging Scalability Pathways

To overcome current limitations, several strategies have been proposed to enhance scalability and industrial viability.

#### 7.3.1. Process Intensification and Bioreactor Design

Advances in bioreactor systems—such as packed-bed and rotating drum reactors—offer potential for improving aeration, mixing, and uniformity in large-scale cultivation [68]. These systems can reduce growth time and improve consistency by controlling environmental conditions more precisely [39].

#### 7.3.2. Standardization and Automation

Automation of inoculation, incubation, and post-processing steps can reduce variability and improve reproducibility. The development of standardized protocols for substrate preparation, growth conditions, and testing is critical for scaling production and ensuring quality control [1].

#### 7.3.3. Hybrid Material Strategies

Incorporating reinforcement materials (e.g., natural fibers, nanocellulose, or mineral fillers) can enhance mechanical performance and reduce reliance on densification alone [6]. Such hybrid systems may enable MBCs to meet performance requirements for broader applications, including semi-structural components.

#### 7.3.4. Modular Manufacturing Approaches

Decentralized production using locally available biomass has been proposed as a scalable model aligned with the principles of the circular economy. This approach reduces transportation costs and leverages regional feedstock availability, although it introduces challenges in maintaining consistent product quality [8].

### 7.4. Pathways Toward Industrial Adoption

For MBCs to achieve widespread industrial adoption, several critical milestones must be addressed:

1. Performance Standardization: Establishing benchmarks for mechanical, thermal, and durability properties;
2. Process Optimization: Reducing growth time and improving densification efficiency;
3. Regulatory Compliance: Meeting safety and building standards for targeted applications;
4. Economic Viability: Achieving cost competitiveness with conventional materials;
5. Life Cycle Integration: Demonstrating environmental benefits through robust life cycle assessment.

Importantly, the pathway to adoption is likely to be application-specific, with low-performance applications (e.g., packaging) serving as entry points before expansion into higher-performance domains.

### 7.5. Critical Outlook

The industrialization of MBCs requires a shift from biologically driven experimentation to engineering-driven optimization. While the biological component enables sustainable material formation, scalability depends on the ability to control and standardize processes in a manner comparable to conventional manufacturing systems.

Future progress will depend on integrating: (i) materials science (substrate engineering), (ii) bioprocess engineering (growth optimization), and (iii) manufacturing technologies (automation and densification).

By addressing these challenges, MBCs can transition from niche applications to mainstream sustainable materials, contributing to the broader goals of the circular economy and low-carbon manufacturing.

## 8. Conclusions

This review advances the understanding of MBCs by integrating fragmented literature into a coherent PSP framework. The analysis demonstrates that MBC performance is governed not by isolated variables, such as fungal species, but by the synergistic effects of other variables: substrate characteristics, growth conditions, and post-processing parameters. A key finding is that densification and moisture control are the dominant determinants of mechanical performance, often outweighing species-specific effects. While fungal growth establishes the initial binding network, pressing and drying ultimately define density, porosity, and load-bearing capacity. This insight resolves inconsistencies across studies and repositions MBCs as engineered biomaterials rather than purely biological systems.

The wide variability in reported properties (<0.1 to >4 MPa compressive strength) is primarily attributed to methodological inconsistencies, including differences in substrate preparation, incubation conditions, and testing protocols. This highlights the urgent need for standardized fabrication and characterization methods to enable reproducibility and meaningful comparison. Substrate properties—particularly the C:N ratio, particle size, and moisture content—are identified as critical design variables influencing fungal metabolism and structural development. Their integration with controlled processing conditions enables targeted property optimization.

From an industrial perspective, MBCs are advancing in packaging and insulation but remain limited in structural applications due to scalability and consistency challenges. Future progress depends on process standardization, hybrid reinforcement strategies, and scalable manufacturing approaches. Overall, this work provides a foundation for predictive design and industrial translation, positioning MBCs as viable, sustainable alternatives in next-generation composite materials.

## Figures and Tables

**Table 1:** Comparison of representative review articles on mycelium-bound composites and the present study.

Review Focus	Fabrication Overview	Species & Substrate Discussion	Application Survey	Quantitative Property Synthesis	PSP Framework	Standardization Roadmap	TRL/Scalability Assessment	Present Study Advantage
Jones review Contributions [2]	✓	✓	Limited	✗	✗	✗	✗	Primarily descriptive review of mycelium materials
Attias review [7]	✓	✓	✓	Limited	✗	✗	✗	Focused on fabrication and sustainability aspects
Appels et al. [1]	✓	Partial	Limited	Limited	✗	✗	✗	Emphasis on material properties and processing
MBC reviews [8,9]	✓	✓	✓	Limited	✗	✗	✗	Broad overview of biofabrication approaches
MBC Reviews (2021–2025)	✓	✓	✓	Partial	✗	Limited	Limited	Primarily descriptive summaries
Present Review	✓	✓	✓	Comprehensive	✓ Proposed PSP Framework	✓ Reporting & Testing Guidelines	✓ Technology Readiness Evaluation	Integrates quantitative synthesis, predictive design framework, standardization roadmap, and industrial translation pathway

Key: ✓ = Included; ✗ = Not explicitly addressed.

**Table 2:** Species-specific white-rot influence on MBCs mechanical performance.

Fungal Species	Substrate	Density (kg/m^3^)	Compressive Strength (MPa)	Key Observation	Reference
*Pleurotus ostreatus*	Sawdust/straw	310–413	0.51	Moderate strength; substrate-dependent	[14]
*Pleurotus ostreatus*	Fiber-reinforced	-	Up to 2.9	Reinforcement + densification critical	[15]
*Ganoderma lucidum*	Beech/sawdust	-	Up to 2.49	Strong binding with densification	[16]
*Trametes versicolor*	Lignocellulosic waste	-	strength-to-weight 0.318 MPa/g	Highest efficiency among species tested	[17]
*Pycnoporus sanguineus*	Pine sawdust	-	higher than *Peniophora* spp.	Species-dependent variation	[18]
*Lentinus sajor-caju*	Corn husk	~200–340	~0.87	Higher density → better strength	[19]
Mixed white-rot species	Various	—	0.03–4.44	Wide variability across studies	[9]

—: data not available.

**Table 4:** Mechanistic comparison between mycelium binding and synthetic adhesives.

Parameter	Mycelium-Based Composites	Synthetic Adhesive Composites	Refs.
Binding mechanism	Hyphal entanglement, enzymatic modification, and hydrogen bonding	Covalent polymer crosslinking (e.g., UF, PF resins)	[1,2,35]
Structural continuity	Discontinuous biological network	Continuous polymer matrix	[1,2]
Variability	High (biological growth variability)	Low (controlled chemistry)	[17,18]
Strength driver	Density, moisture, pressing conditions	Resin chemistry, curing conditions	[17,35]
Moisture sensitivity	High	Moderate–low	[18,35]
Biodegradability	High	Low (petrochemical-based)	[1,2]
Industrial maturity	Emerging (TRL 3–6)	Fully mature (TRL 9)	[2]

**Table 5:** Carbon-to-nitrogen (C:N) ratios of common substrates used in mycelium-based composites.

Substrate Type	Sample Substrates	C:N Ratio	Notes on Relevance to MBCs	References
Agricultural residues (cereal-based)	Wheat straw, rice straw	80:1–100:1	High lignocellulose content supports structural integrity but often requires nitrogen supplementation to enhance mycelial growth	[45,46]
Maize residues	Corn stover, corn husk	90:1–120:1	Coarse fibrous matrix enhances porosity; excessive C:N may limit colonization without nutrient amendment	[5,45]
Wood-based substrates	Hardwood sawdust, wood chips	200:1–500:1	Excellent structural scaffold; very low nitrogen slows growth unless blended with nutrient-rich substrates	[3]
Fibrous bast materials	Hemp hurds, flax shives	60:1–90:1	Balanced fiber morphology promotes strong hyphal binding and improved mechanical performance	[47]
Coconut-based residues	Coconut coir, husk fiber	70:1–100:1	High lignin improves water resistance; moderate enzymatic accessibility	[46]
Agro-industrial by-products	Brewer’s spent grain, wheat bran	15:1–25:1	Nitrogen-rich substrates accelerate growth but may reduce composite stiffness if used alone	[5,38]
Oilseed processing residues	Rapeseed cake, soybean meal	8:1–20:1	High protein content enhances colonization; typically used as supplements rather than bulk substrate	[4]
Distillers dried grains with solubles (DDGS)	Corn-based DDGS	10:1–25:1	Effective nitrogen supplement; excessive use may weaken composite cohesion without lignocellulosic blending	[48]
Optimal blended substrates (target range)	Mixed lignocellulose + nutrient supplement	20:1–30:1	Reported as optimal for balanced mycelial metabolism and composite performance	[5]

**Table 6:** Variability in substrate preparation across studies.

Parameter	Common Practice	Reported Variability	Impact on Properties	Refs.
Substrate type	Sawdust, straw	Highly diverse	Affects density, strength	[1,5]
Particle size	Mixed fractions	0.5–10 mm	Controls porosity & bonding	[1]
Pre-treatment	Sterilization	Pasteurization/none	Influences contamination & growth	[2]
Supplementation	Bran, DDGS	Variable ratios	Alters C:N balance & growth	[40]

**Table 7:** Growth and incubation parameters across studies.

Parameter	Typical Range	Variability	Effect on MBC
Temperature	20–30 °C	Moderate	Growth rate
Incubation time	5–21 days	High	Network formation
Inoculum loading	5–20%	Moderate	Colonization uniformity
Humidity	70–95%	High	Moisture balance

**Table 8:** Post-processing variability and its effects.

Parameter	Range	Effect on Structure	Effect on Properties	Ref.
Drying temp	25–80 °C	Shrinkage, cracking	Stability ↑	[3]
Pressing pressure	0–2 MPa	Density increase	Strength ↑	[1]
Drying time	Hours–days	Moisture removal	Durability ↑	[2]

**Table 9:** Testing and reporting disparities.

Parameter	Variability	Consequence
Sample size	Non-standard	Scale-dependent strength
Loading rate	Not uniform	Affects measured strength
Moisture conditioning	Often omitted	Alters stiffness
Reporting metrics	Inconsistent	Poor comparability

**Table 10:** The literatures that demonstrate agreement between PSP predictions and experimentally observed outcomes.

Process Variable	Structural Modification	Predicted Property Outcome	References
Mechanical pressing	Reduced porosity; increased density	Higher compressive strength and stiffness	[1,5]
Fiber reinforcement	Enhanced load transfer pathways	Increased strength and modulus	[4]
Smaller particle size	Greater packing efficiency	Higher density and compressive strength	[3,62]
Balanced C:N ratio	Uniform colonization and bonding	Improved structural cohesion	[6,45,46]
Excessive moisture content	Increased voids and contamination risk	Reduced mechanical performance	[1,7]
Heat-assisted pressing	Lignin softening and consolidation	Enhanced water resistance and strength	[1,51]

## Data Availability

No new data were created or analyzed in this study. Data sharing does not apply to this article.

## Abbreviations

The following abbreviations are used in this manuscript:

MBCs	Mycelium-bound Composites
TRL	Technology Readiness Level
MDF	Medium-Density Fiberboard
UF	Urea Fiberboard
PSP	Process–Structure–Property
